# RNA and phosphoprotein profiles of TP53- and PTEN-knockouts in MCF10A at baseline and responding to DNA damage

**DOI:** 10.1038/s41597-023-02829-1

**Published:** 2024-01-04

**Authors:** ChenWei Lin, Regine M. Schoenherr, Uliana J. Voytovich, Richard G. Ivey, Jacob J. Kennedy, Jeffrey R. Whiteaker, Pei Wang, Amanda G. Paulovich

**Affiliations:** 1 Fred Hutchinson Cancer Center, Seattle, WA USA; 2 Department of Genetic and Genomic Sciences, Icahn School of Medicine at Mount Sinai, New York, NY USA

**Keywords:** Breast cancer, Breast cancer

## Abstract

A wealth of proteogenomic data has been generated using cancer samples to deepen our understanding of the mechanisms of cancer and how biological networks are altered in association with somatic mutation of tumor suppressor genes, such as TP53 and PTEN. To generate functional signatures of TP53 or PTEN loss, we profiled the RNA and phosphoproteomes of the MCF10A epithelial cell line, along with its congenic TP53- or PTEN-knockout derivatives, upon perturbation with the monofunctional DNA alkylating agent methyl methanesulfonate (MMS) vs. mock treatment. To enable quantitative and reproducible mass spectrometry data generation, the cell lines were SILAC-labeled (stable isotope labeling with amino acids in cell culture), and the experimental design included label swapping and biological replicates. All data are publicly available and may be used to advance our understanding of the TP53 and PTEN tumor suppressor genes and to provide functional signatures for bioinformatic analyses of proteogenomic datasets.

## Background & Summary

The DNA damage response (DDR) network is a complex system of pathways that acts as an anti-cancer barrier in early human tumorigenesis. Defects in the DDR network are highly associated with carcinogenesis and tumor progression. Furthermore, the DDR network is constitutively activated in early-stage cancers, compared to normal epithelium^1,2^. Constitutive activation of oncogenes can lead to increased replication stress, and to the formation of DNA double-strand breaks^3^ that activate the ATM/ATR-dependent DDR^4^. Because the DDR network includes multiple mechanisms of activation, additional mutations (e.g., TP53) may enable tumors to circumvent this mechanism and advance to acquire increasingly more malignant properties.

Both TP53 and PTEN are mutated in breast cancers, and both are connected to the DDR^5–7^. To add to the knowledge of the effects of TP53 and PTEN mutations on the DDR network, we performed RNA-seq and phosphoproteomic profiling of three congenic cell lines (MCF10A, MCF10A TP53-knockout (KO), and MCF10A PTEN-KO) following mock treatment or exposure to the monofunctional DNA alkylating agent methyl methanesulfonate (MMS). Alkylating agents, commonly used in cancer chemotherapy, are known to induce replication stress, potentially mimicking the activation of the DDR network observed in early-stage human breast cancers.

Our goals were to determine functional signatures of TP53 and PTEN mutations (in the presence or absence of an activated DDR), to add to our knowledgebase of the biological effects of these mutations, and to provide empirical functional signatures^8–11^ associated with these mutations, to aid in the bioinformatic analysis of proteogenomic profiles (for example).

## Methods

### Experiment design

**Fig. 1:**
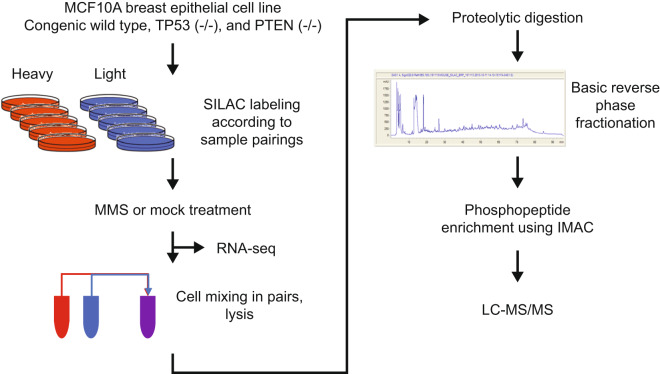
Sample processing workflow for phosphoproteomic and RNA-seq data generation. Three congenic cell lines were paired for SILAC labeling as given in Table 1 and subsequently treated using the DNA alkylating agent methyl methanesulfonate (MMS), or mock-treated. The samples were analyzed either by RNA-seq, or were further processed for phosphoproteomic analysis by mixing the cells of the SILAC pairs, lysing the cells, and enzymatically digesting the lysates using Lys-C and trypsin. The digests were fractionated using basic (high pH) reverse phase liquid chromatography and phosphopeptides were enriched by immobilized metal affinity chromatography (IMAC). The final phosphoproteomic samples were analyzed by LC-MS/MS on an LTQ-Orbitrap Elite mass spectrometer.

Our goal was to study how phosphoproteins and gene expressions in the wild type MCF10A epithelial cell line, along with congenic TP53- or PTEN-knockout derivatives, may change in response to MMS perturbations. An overview of the experimental workflow is shown in Fig. 1, and summaries of all samples that were generated for the phosphoproteomic and genomic analyses are shown in Tables 1, 2.

**Table 1:** Summary of the phosphoproteomic dataset results for the SILAC-labeled phosphoproteomic samples and label-swap pairs analyzed by LC-MS/MS.

Expt #	Heavy labeling	Light labeling	Labeling experiment	Unique proteins	Unique phospho sites	pY containing peptides	pS containing peptides	pT containing peptides
*1*	*MCF10A-WT-MMS*	*MCF10A-WT-Mock*	*Forward*	*2548*	*7958*	*105*	*7210*	*1342*
***2***	***MCF10A-WT-MMS***	***MCF10A-WT-Mock***	***Forward***	***2391***	***7053***	***118***	***6385***	***1070***
**3**	**MCF10A-WT-Mock**	**MCF10A-WT-MMS**	**Reverse**	**2726**	**8831**	**113**	**8020**	**1463**
**4**	**MCF10A-TP53-KO-MMS**	**MCF10A-TP53-KO-Mock**	**Forward**	**2803**	**8996**	**102**	**8203**	**1348**
**5**	**MCF10A-TP53-KO-Mock**	**MCF10A-TP53-KO-MMS**	**Reverse**	**2302**	**6680**	**77**	**6100**	**958**
6	MCF10A-TP53-KO-MMS	MCF10A-WT-MMS	Forward	2723	8715	92	7945	1282
7	MCF10A-TP53-KO-Mock	MCF10A-WT-Mock	Forward	2708	8744	80	7986	1352
**8**	**MCF10A-PTEN-KO-MMS**	**MCF10A-PTEN-KO-Mock**	**Forward**	**2383**	**7266**	**86**	**6611**	**1093**
**9**	**MCF10A-PTEN-KO-Mock**	**MCF10A-PTEN-KO-MMS**	**Reverse**	**2311**	**6479**	**79**	**5902**	**926**
10	MCF10A-PTEN-KO-MMS	MCF10A-WT-MMS	Forward	2172	5925	79	5440	840
11	MCF10A-PTEN-KO-Mock	MCF10A-WT-Mock	Forward	2483	7347	95	6691	1140
**Overall unique proteins or phosphosites/phosphopeptides:**				**4200**	**21740**	**582**	**19356**	**4532**

The experiments indicated in bold (Expts 2-3, 4-5, and 8-9) indicate heavy and light label-swap SILAC pairs. *Italic* font indicates the two biological replicate experiments (Expts 1-2). Only phosphosites and phosphopeptides with phosphosite localization scores >0.8 were included in the table. For the pY, pS, and pT columns, peptides were counted in two columns if a peptide sequence contained, for example, both a pS and a pT phosphorylation; there were 2345 phosphopeptides that were counted in two columns. KO: knockout; MMS: methyl methanesulfonate-treated; WT: wild type.

**Table 2:** Summary of RNA-seq results.

Sample	Biological replicate number	Raw read numbers	Read numbers after alignment	Read numbers after filtering	Number of genes identified
MCF10A-WT-Mock	1	30,072,593	29,832,012	14,604,587	17012
	2	33,586,288	33,317,598	16,272,759	16774
	3	24,664,555	24,467,239	11,953,880	17087
MCF10A-WT-MMS	1	31,334,597	31,083,920	15,179,500	17477
	2	33,368,051	33,101,107	16,219,822	17409
	3	34,559,225	34,282,751	16,799,741	17479
MCF10A-PTEN-KO-Mock	1	28,813,360	28,611,666	14,022,122	16870
	2	29,943,135	29,703,590	14,555,672	16762
	3	29,276,884	29,042,669	14,208,258	16747
MCF10A-PTEN-KO-MMS	1	27,801,146	27,578,737	13,467,010	17154
	2	25,186,687	24,985,194	12,218,955	17130
	3	28,697,538	28,467,958	13,915,742	17377
MCF10A-TP53-KO-Mock	1	27,776,335	27,581,901	13,528,363	17045
	2	27,333,676	27,115,007	13,242,834	17149
	3	24,866,216	24,667,286	12,072,204	16890
MCF10A-TP53-KO-MMS	1	29,671,537	29,434,165	14,366,235	17394
	2	25,917,750	25,710,408	12,583,207	17509
	3	31,102,027	30,853,211	15,103,736	17405

Three biological replicates were prepared for each cell line and treatment on 3 independent days. KO: knockout; MMS: methyl methanesulfonate-treated; WT: wild type.

Specifically, to allow for comparisons of phosphoprotein levels between MMS and mock treatment, and between the different cell lines, pairs of cultured cells were metabolically labeled by stable isotope labeling with amino acids in cell culture (SILAC)^12,13^. In a SILAC experiment, one cell population is grown in a medium containing natural ^12^C6;^14^N2-lysine and ^12^C6;^14^N4-arginine, and another in a medium containing heavy isotopes ^13^C6;^15^N2-lysine and ^13^C6;^15^N4-arginine. When the two populations are mixed and analyzed by mass spectrometry, peptides stemming from the two populations can be distinguished by their different mass-to-charge ratios, and the relative peak intensities reflect the abundance ratios. In total, 11 pairs of samples were profiled by LC-MS/MS (Table 1).

In the RNA-seq study, three biological replicates (prepared on three independent days) were used for each cell-line and treatment group, and 18 RNA-seq profiles were generated (Table 2).

### Cell culturing and processing

The non-tumorigenic MCF10A epithelial cell line derived from adherent cells in the breast tissue/mammary gland was purchased from Sigma-Aldrich (Sigma-Aldrich, CLL1040-1VL). A zinc finger nuclease (ZFN) knockout corresponding to a TP53 deletion (Sigma-Aldrich, CLLS1049) and a ZFN knockout corresponding to a PTEN deletion (Sigma-Aldrich, CLLS1046) in MCF10A cells were also purchased from Sigma-Aldrich. All cell line identities were confirmed by DNA fingerprinting using STR (Short Tandem Repeats) CODIS (Combined DNA Index System) typing. The cell lines were maintained at 37 °C in 5% CO_2_ and cultured in DMEM/F-12 medium (Gibco, 11320) supplemented with 5% horse serum (Gibco, 16050), cholera toxin (Sigma-Aldrich, C8052) to a final concentration of 1 ng/mL, insulin (Sigma-Aldrich, I6634) to a final concentration of 10 μg/mL, human epidermal growth factor (PeproTech, AF-100-15) to a final concentration of 10 ng/mL, hydrocortisone (Sigma-Aldrich, C8052) to a final concentration of 0.5 μg/mL, and 1% Pen Strep (Gibco, 15140).

For the differential isotopic labeling of cells for SILAC analysis, Dulbecco’s Modified Eagle Medium/Nutrient Mixture F-12 (DMEM:F-12) SILAC medium deficient in both L-lysine and L-arginine supplemented with heavy or light amino acids was used. Heavy SILAC growth medium consisted of DMEM:F-12 for SILAC (Thermo, 88370) containing ^13^C6;^15^N2 lysine (Cambridge Isotope Laboratories, CNLM-291-H-0.1) and ^13^C6;^15^N4 arginine (Cambridge Isotope Laboratories, CNLM-537-H-0.1) with growth supplements containing 5% dialyzed horse serum (Valley Biomedical, AS3053), cholera toxin to a final concentration of 1 ng/mL, insulin to a final concentration of 10 μg/mL, human epidermal growth factor to a final concentration of 10 ng/mL, hydrocortisone to a final concentration of 0.5 μg/mL, and 1% Pen Strep. Light SILAC growth medium consisted of DMEM:F-12 for SILAC containing unlabeled lysine (Cambridge Isotope Laboratories, ULM-8766-0.1) and unlabeled arginine (Cambridge Isotope Laboratories, ULM-8347-0.1) with growth supplements containing 5% dialyzed horse serum, cholera toxin to a final concentration of 1 ng/mL, insulin to a final concentration of 10 μg/mL, human epidermal growth factor to a final concentration of 10 ng/mL, hydrocortisone to a final concentration of 0.5 μg/mL, and 1% Pen Strep. The cell lines were cultured in Heavy SILAC growth medium or Light SILAC growth medium at a minimum of three passages to ensure incorporation of heavy or light amino acids.

Two days prior to cell line lysis, cells were plated in 100 mm culture dishes using an equal number of cells per dish (example: 1 million cells per 100 mm culture dish). 48 hours later, the growth medium was replaced with heavy or light growth medium containing 0.5 mM of MMS (Sigma-Aldrich, 129925) or heavy or light growth medium containing no MMS (mock treatment). The cells were incubated for 3 hours at 37 °C in 5% CO_2_. At the end of the incubation time, the growth medium was removed, and the adherent cells were rinsed with DPBS (Gibco, 14190). The cells were detached using 0.25% Trypsin-EDTA (Gibco, 25200) and placed in the incubator at 37 °C in 5% CO_2_ for 15–20 minutes. The Trypsin-EDTA solution was inactivated by adding Trypsin Neutralization Solution (TNS, DMEM:F-12 SILAC media containing 5% dialyzed horse serum) and the remaining attached cells were scraped off the plate with a cell scraper. The cells were transferred to pre-cooled 50 mL conical tubes, spun at 400 × g for 8 min at 4 °C to remove the medium, and washed twice with ice-cold DPBS. Cells for RNA-seq analysis were further treated as described in the **RNA-seq sample preparation** section below. For LC-MS/MS analysis, freshly-prepared ice-cold urea lysis buffer (containing 6 M urea (Sigma-Aldrich, U0631), 25 mM Tris (pH 8.0) (Sigma-Aldrich, T2194), 1 mM EDTA (Sigma-Aldrich, E7889), 1 mM EGTA (Sigma-Aldrich, E0396), 1% phosphatase inhibitor cocktail 2 (Sigma-Aldrich, P5726), 1% phosphatase inhibitor cocktail 3 (Sigma-Aldrich, P0044), and 1% protease inhibitor cocktail (Sigma-Aldrich, P3840)) was added to cell pellets at a concentration of 25 million cells per 1 mL of urea lysis buffer. The cell lysate suspension was sonicated twice for 15 seconds using a Sonic Dismembrator (Fisher Scientific, Model 100) at setting level 1 and placed on ice for 30 seconds between sonications. The lysates were transferred to microcentrifuge tubes, vortexed, and then cleared by centrifugation at 20,000 × g for 10 min at 4 °C. Supernatants were transferred to cryo-vials and stored in liquid nitrogen until ready for use.

### Western blotting

Protein lysates (50 μg/lane) were resolved by SDS PAGE on 4–12% Bis-Tris Novex gels (Thermo Fisher) and transferred to 0.45-μm nitrocellulose membranes using an XCell II™ Blot Module (Thermo Fisher). Membranes were blocked for 1 h in SuperBlock (Pierce) with 0.1% Tween 20 (Sigma) and primary antibody (α-p53 (Epitomics, 1026-1), α-PTEN (Epitomics, 5171-1), or α-alpha Tubulin (Epitomics, 1878-1)) was incubated overnight at 4 °C (a separate Western blot was used for the alpha Tubulin loading controls). Membranes were washed two times with PBS, 0.1% Tween 20. HRP-conjugated goat anti-rabbit secondary antibody (Cell Signaling Technology (CST), 7074) diluted 1:2000 in 1x PBS, 10% SuperBlock, and 0.1% Tween 20 was added to the membrane and incubated 1 hour at room temperature. Membranes were washed two times with PBS, 0.1% Tween 20 and antibody was visualized with 1 × LumiGLO substrate (CST, 7003).

### Protein digestion

Protein in lysates was quantified by Micro BCA Assay (ThermoFisher, 23235), and heavy lysate samples were mixed with light lysate samples 1:1 based on protein mass and subsequently diluted to 5 mg/mL using lysis buffer. The lysates were reduced in 76 mM TCEP (ThermoFisher, 77720) for 30 minutes at 37 °C with shaking, followed by alkylation with 134 mM iodoacetamide (Sigma, A3221-10VL) in the dark at room temperature for 30 minutes. Lysates were then diluted with 1.2 mL 200 mM Tris (pH 8.0). Lys-C (Wako, 129-02541) was dissolved in 25 mM Tris (pH 8.0) at 200 μg/mL and added to lysates at 1:100 (enzyme:protein) ratio by mass and incubated for 2 hours at 37 °C with shaking. Trypsin (Promega, V5113) was then added at a 1:50 trypsin:protein ratio and incubated for 2 hours at 37 °C with shaking. After 2 hours, a second trypsin aliquot was added at a 1:100 trypsin:protein ratio. Digestion was carried out overnight at 37 °C with shaking. After 16 hours, the reaction was quenched with formic acid (FA, EMD Millipore, 1.11670.1000) to a 1% final concentration by volume. Samples were desalted using Oasis HLB 96-well plates (Waters) and a positive pressure manifold (Waters). The plate wells were washed with 3 × 400 μL of 50% acetonitrile (MeCN, Fisher Scientific, A955-4)/0.1% FA, and then equilibrated with 4 × 400 μL of 0.1% FA. The digests were applied to the wells, then washed with 4 × 400 μL 0.1% FA before being eluted drop by drop with 3 × 400 μL of 50% MeCN/0.1% FA. The eluates were lyophilized, followed by storage at −80 °C until use.

### Basic (high pH) reverse phase (RP) liquid chromatography and immobilized metal affinity chromatography (IMAC)

The desalted tryptic digest (4 mg) was fractionated by high-pH reverse phase (RP) liquid chromatography as described previously^14^ to generate 12 samples, which were dried down and stored at −80 °C prior to phosphopeptide enrichment. Immobilized metal affinity chromatography (IMAC) enrichment was performed using Ni-NTA-agarose beads (Qiagen, 36113) prepared as Fe3 + -NTA-agarose beads as described previously^15^ with the following changes. Peptide enrichment was performed on fractionated lysate digest reconstituted in 500 μL of 0.1% Trifluoroacetic Acid (TFA, Thermo, 28901) in 80% MeCN and incubated for 30 minutes with 300 μL of the 5% bead suspension, mixing at 1400 rpm at room temperature. After incubation, the beads were washed 3 times with 150 μL of 0.1% TFA in 80% MeCN. Phosphorylated peptides were eluted 2 times from the beads using 150 μL of 500 mM Potassium Phosphate, pH 7 (Fisher, S80146-3, S80146-1) for 1 minute with agitation at room temperature (to not exceed 5 min). Samples were desalted by StageTip (Thermo Scientific, SP301). The StageTips were first equilibrated by the following 20 μL additions, followed by centrifugation at 2,000 × g for 1 minute: MeOH, 0.1% FA in 50% MeCN, 2 × 1% FA. Samples were loaded onto the StageTips in 2 × 150 μL additions followed by centrifugation at 2,000 × g for 1 minute. The samples were washed 2x with 40 μL of 1% FA followed by centrifugation at 2,000 × g for 1 minute and eluted with 40 μL of 0.1% FA in 50% MeCN followed by centrifugation at 2,000 × g for 1 minute. The eluate was dried down and re-suspended in 0.1% FA, 3% MeCN. The samples were frozen at −80 °C until analysis.

### Nano-liquid chromatography-tandem mass spectrometry

Phosphopeptide-enriched samples were analyzed by LC-MS/MS on an Easy-nLC 1000 (Thermo Scientific) coupled to an LTQ-Orbitrap Elite mass spectrometer (Thermo Scientific) operated in positive ion mode. The LC system, configured in a vented format, consisted of a fused-silica nanospray needle (PicoTip™ emitter, 50 µm ID × 20 cm, New Objective) packed in-house with Magic C18-AQ, 5 µm and a trap (IntegraFrit™ Capillary, 100 µm ID × 2 cm, New Objective) containing the same resin as the analytical column with mobile phases of 0.1% FA in water (A) and 0.1% FA in MeCN (B). The peptide sample was diluted in 20 µL of 0.1% FA, 2% MeCN and 8.5 µL was loaded onto the column and separated over 150 minutes at a flow rate of 300 nL/min with a gradient from 5 to 7% B for 2 min, 7 to 35% B for 150 min, 35 to 50% B for 1 min, hold 50% B for 9 min, 50 to 95% B for 2 min, hold 95% B for 7 min, 95 to 5% B for 1 min, re-equilibrate at 5% B for 1 min. A spray voltage of 2000 V was applied to the nanospray tip. MS/MS analysis consisted of 1 full scan MS from 400–1800 m/z at resolution 120,000 followed by data dependent MS/MS scans using 35% normalized collision energy of the 20 most abundant ions. Selected ions were dynamically excluded for 30 seconds.

### Shotgun mass spectrometry data analysis

Raw MS/MS spectra from the analysis were searched against the UniProt database UP000005640_9606_human (UniProt release 2019_10) using MaxQuant/Andromeda (MaxQuant_1.6.10.43)^16^. The search was performed with the tryptic enzyme constraint set for up to two missed cleavages, oxidized methionine and phosphorylated serine, threonine, and tyrosine set as variable modifications, and carbamidomethylated cysteine set as a static modification. Multiplicity was set at 2, with 3 maximum labels, with Arg10 and Lys8 selected as heavy labels. Peptide MH + mass tolerances were set at 20 ppm. The overall FDR was set at ≤1%. Any phosphosite localization with a probability greater than 0.8 was deemed as being localized; below that was deemed as an ambiguous localization. All figures and the tables including phosphoproteomic data are based on phosphopeptides having phosphosite localization scores >0.8. Quantification of Heavy:Light ratios was performed by MaxQuant. The MaxQuant results are provided in the ‘MaxQuant output for SILAC experiments’ Table in ‘Data and Results Summary Tables’ (data are at figshare)^17^.

Specifically, phosphopeptides and their corresponding phosphoproteins were considered differentially expressed if their heavy-to-light SILAC ratios were ≥2 or ≤0.5 in the label swap experiments between mock- and MMS-treated samples (as highlighted in the ‘134 Phosphopeptides’ Table in ‘Data and Results Summary Tables’ (data are at figshare)^17^). Moreover, in the Technical Validation section, we further evaluated the CV of the SILAC ratios based on the replicate pairs of experiments (experiments 2 and 3, 4 and 5, and 8 and 9 in Table 1).

### RNA-seq sample preparation

Total RNA was extracted from cells treated with or without MMS in light SILAC growth medium for 3 hours using the RNeasy Mini Kit (Qiagen, 74104) coupled with the QIAshredder homogenizers (Qiagen, 79654). RNA quality was assessed using an Agilent 2100 Bioanalyzer and RNA was only accepted if the RNA Integrity Number (RIN) was > 9.0. 1.0 μg of total RNA from each sample was then polyA selected and chemically fragmented to ∼200 bp, and cDNA was created using random hexamer primers. Library preparation followed the TruSeq Illumina protocol with each individual library receiving a unique Illumina barcode. RNA-seq was performed on an Illumina HiSeq 2500 machine with six libraries multiplexed per lane using 50-bp paired-end reads. This resulted in an average of 250 million reads per lane, with an average of 43 million reads per sample. Each sample had three biological replicates that were prepared on three separate days.

### RNA sequencing and data analysis

The transcripts from all cell line samples were reassembled using human reference genome UCSC hg19. The pair-end reads were aligned using TopHat version 1.1.4 and two mismatches in the alignment were allowed. We obtained a high mapping rate with 77–83% of reads mapped to the reference genome and 67% were uniquely mapped. Paired-end reads were properly trimmed and filtered by Cutadapt (v.1.12), and only reads with a Phred quality score >20 and read length >50 bp were used in subsequent analysis^18^. All RNA-seq samples passed FastQC’s basic statistics test. The gene level read counts data were normalized as counts per million (CPM) using the R package edgeR with trimmed mean of M-values normalization (TMM) method^19^ to adjust for sequencing library size differences.

We then documented expression changes due to genetic and/or chemical perturbations based on linear regression analysis. Specifically, we used the regression below (R package *glm* with Gaussian distribution) to jointly model RNA-seq profiles of different cell lines under different perturbations:

$$\log 2\left({\rm{CPM}}\right) \sim {\rm{tp}}53+{\rm{pten}}+{\rm{mms}}+{\rm{tp}}5{3}^{* }{\rm{mms}}+{{\rm{pten}}}^{* }{\rm{mms,}}$$

where *tp53*, *pten*, and *mms* are indicators for either the mutation or treatment status. We chose this analysis since our RNA-seq experiment used a two-factor factorial design. The first factor is the genetic “mutation” status: wild type, PTEN-KO and TP53-KO; and the second factor is the treatment status: mock vs. MMS treatment. Thus, we employed the multiple regression model to better account for both the marginal and the interaction effects of these factors. Specifically, the regression model includes three main effect terms for PTEN-KO, TP53-KO, and MMS treatment; and two interaction effect terms for PTEN-KO × MMS and TP53-KO × MMS. The results are provided in the ‘GLM output’ Table in ‘Data and Results Summary Tables’ (data are at figshare)^17^.

## Data Records

### Raw data files

The mass spectrometry proteomics data have been deposited to the ProteomeXchange Consortium via the PRIDE^20^ partner repository with the dataset identifier PXD028494^16^. The uploaded data include 169 raw files, a folder containing the results and details of the database search (all raw data were searched together), and a folder with details from the Andromeda search.

The raw and processed RNA-seq data have been deposited in NCBI’s Gene Expression Omnibus (GEO)^18,21^. The uploaded data include 36 RNA-seq fastq files (paired end) and an associated MCF10A_exons.cpm.gct file which contains a Counts per Million transcripts (CPM) matrix for genes of every sample (see also the ‘RNA-seq gene data’ Table in ‘Data and Results Summary Tables’ (data are at figshare)^17^).

### Processed data files

For the phosphoproteomics data analysis, only phosphosites with localization scores >0.8 were included. A total of 4200 unique phosphoproteins containing 21740 phosphosites were accurately quantified in at least one sample (Table 1 and the ‘MaxQuant output for SILAC experiments’ Table in ‘Data and Results Summary Tables’ (data are at figshare)^17^). Between 2172 to 2803 phosphoproteins (mean, 2505) and 5925 to 8996 phosphosites (mean, 7636) were identified per experiment. 1581 phosphopeptides corresponding to 914 phosphoproteins had no missing values across all experiments. The number of post-translational phosphorylations on serine, threonine, and tyrosine residues for each experiment are included in the Table 1.

**Fig. 2:**
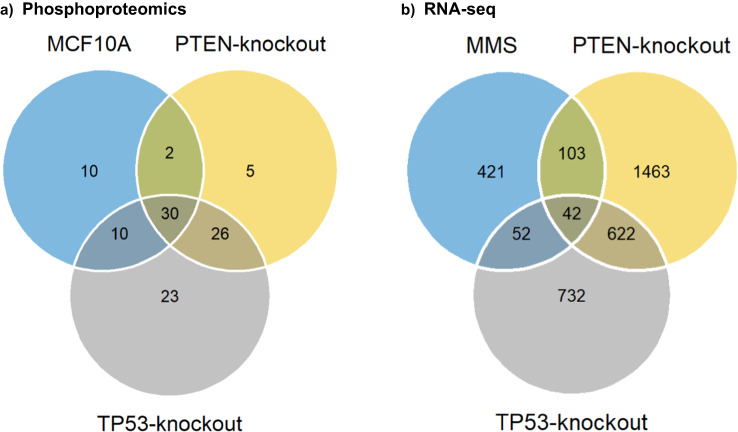
(**a**) Venn diagram depicting the overlap of differentially detected phosphoproteins (with a ≥ 2 or ≤ 0.5 ratio change upon MMS perturbation) between the three forward and reverse SILAC pairs (experiments 2-3, 4-5, and 8-9 in Table 1). (**b**) Venn diagram summarizing the overlap of RNA-seq genes when analyzing the main effects of MMS, the TP53-knockout, and the PTEN-knockout. The genes given in the Venn diagram had an FDR < 0.05 and > 2-fold difference between MMS vs. Mock, MCF10A vs. TP53-knockout, and MCF10A vs. PTEN-knockout.

The phosphoproteomics experiments were designed to allow pairwise and higher-level comparisons between the genetic and chemical perturbations. For example, 2136 phosphopeptides (1147 phosphoproteins) were detected in all six experiments of the three forward and reverse SILAC pairs (experiments 2–3, 4–5, and 8–9 in Table 1 and Table ‘2136 Phosphopeptides’ in ‘Data and Results Summary Tables’ (data are at figshare)^17^), among which, 134 phosphopeptides (corresponding to 106 unique proteins) were identified as having a ≥ 2 or ≤ 0.5 ratio change upon MMS perturbation in the three cell lines (Fig. 2a and Table ‘134 Phosphopeptides’ in ‘Data and Results Summary Tables’ (data are at figshare)^17^). Specifically, 52, 89, and 63 phosphoproteins were differentially expressed between the mock and MMS treatment groups for the MCF10A wild type, TP53-knockout, and PTEN-knockout cell lines, respectively, while 30 phosphoproteins were differentially expressed upon MMS treatment in all three cell lines (see the ‘30 Phosphoproteins’ Table in ‘Data and Results Summary Tables’ (data are at figshare)^17^). Experiments that allow comparisons between the knockout and wild type cell lines, with or without MMS treatment, were also performed (Table 1).

**Fig. 3:**
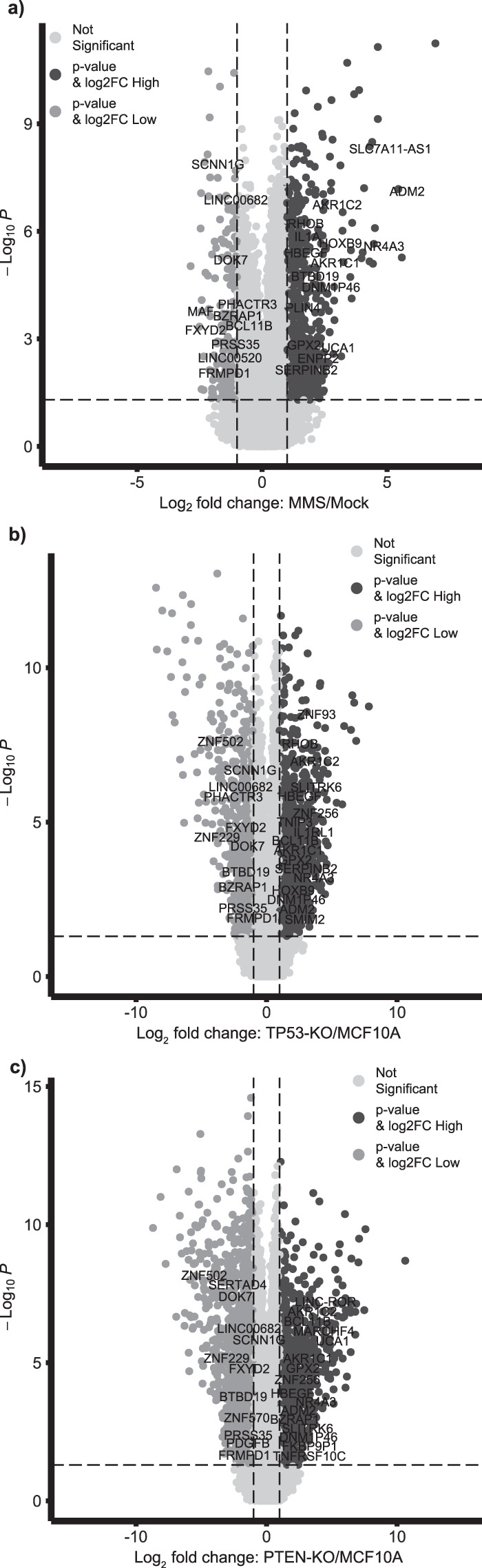
Volcano plots showing the adjusted P-values and the log2 fold change (FC) values of RNA-seq genes for the mock vs. MMS analysis in MCF10A cells (**a**), for the MCF10A vs. TP53-knockout (KO) analysis (**b**), and for the MCF10A vs. PTEN-knockout (KO) analysis (**c**). The 42 genes that were identified as having differential expression due to the three main effects (MMS, TP53-knockout, and PTEN-knockout, Fig. 2b) are labeled in the volcano plots.

The RNA-seq experiments identified a total of 21090 genes (see the ‘RNA-seq gene data’ Table in ‘Data and Results Summary Tables’ (data are at figshare)^17^), and 14458 genes were detected in all samples. A summary of the RNA-seq QC measurements (read numbers) as well as the number of genes observed in each experiment (ranging from 16747 to 17509, with a mean at 17148) is listed in Table 2. Based on the RNA-seq data, we detected a large number of genes differentially expressed due to the genetic and/or chemical perturbations. The subset with FDR < 0.05 and Fold-Change > 2 is summarized in Figs. 2b, 3, and the ‘Venn diagram RNA-seq genes’ Table in ‘Data and Results Summary Tables’ (data are at figshare)^17^. Specifically, 42 genes were identified to be differentially expressed in all perturbations examined, including MMS, PTEN-knockout, and TP53-knockout (Fig. 2b and the ‘42 Genes’ Table in ‘Data and Results Summary Tables’ (data are at figshare)^17^). On the other hand, 421, 732, and 1463 genes were differentially expressed only upon MMS treatment, TP53-knockout, and PTEN-knockout, respectively (Fig. 2b and Table ‘Venn diagram RNA-seq genes’ in ‘Data and Results Summary Tables’ (data are at figshare)^17^). We explored the RNA-seq data further by characterizing the cell line-specific MMS signatures. However, for each cell line, there were only three biological samples in each treatment group (MMS or mock), and hence the power to perform a genome wide screening for MMS signatures based on this small sample size was limited. When we compared the gene expression profiles after MMS treatment (n = 3) vs. those from the Mock group (n = 3) using a t-test, we could not detect any significant differentially expressed genes (FDR < 0.05 & Fold-Change > 2 or < 0.5) for any of the three cell-lines (WT, TP53-KO, and PTEN-KO), see also the ‘Cell line specific gene expression t-tests’ Table in ‘Data and Results Summary Tables’ (data are at figshare)^17^.

## Technical Validation

**Fig. 4:**
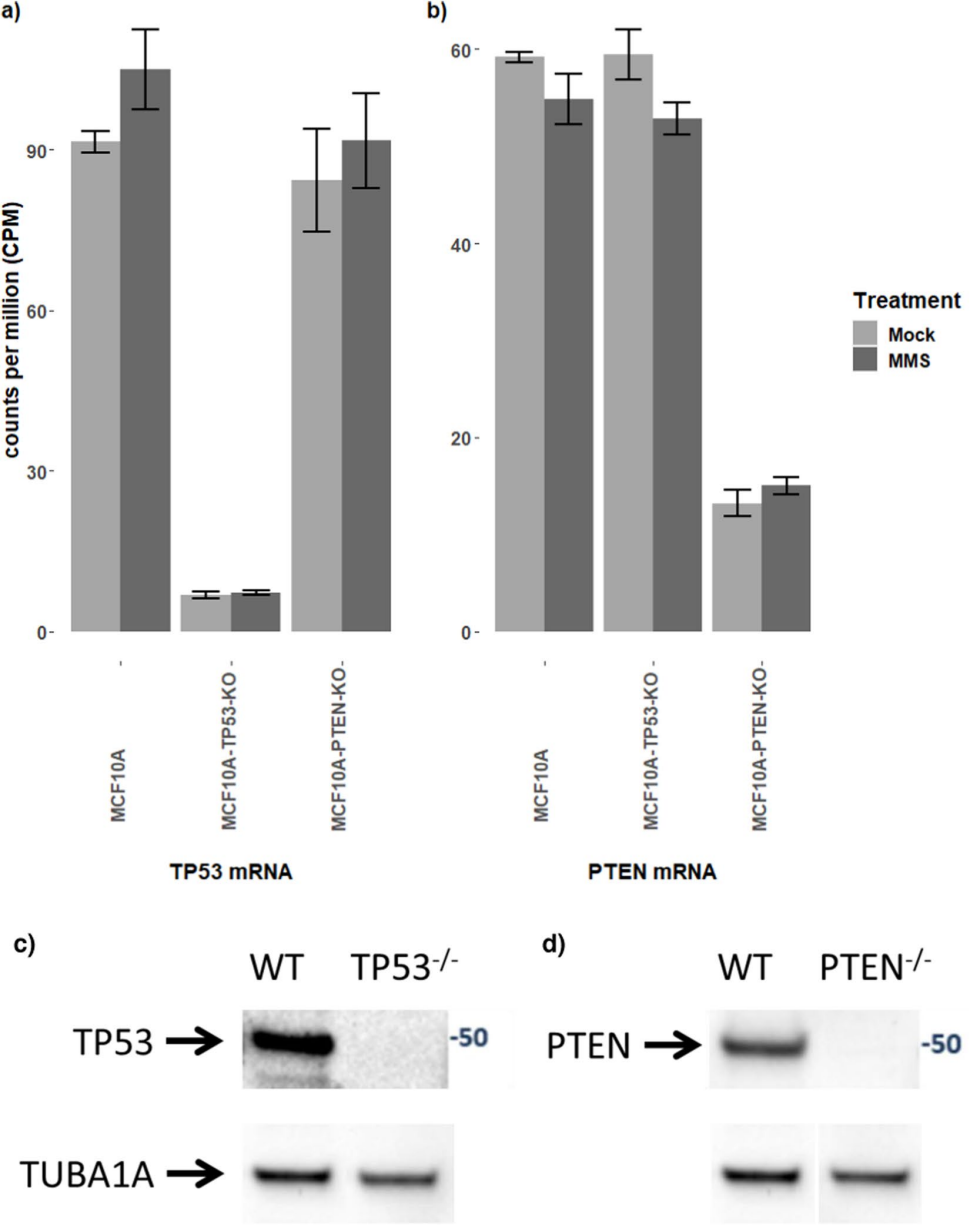
RNA-seq gene and protein expression levels in the wild type MCF10A and TP53- and PTEN-knockout MCF10A cells. TP53 (**a**) and PTEN (**b**) RNA-seq gene expression levels in the different cell lines treated with MMS or mock treatment. Error bars indicate the standard deviation based on three biological replicates. p53 **(c)** and PTEN **(d)** protein expression in the wild type and knockout cell lines tested by western blot.

We employed various controls in our experiments to ensure the technical and biological reproducibility of the dataset and to enable a robust statistical characterization of the effects of the genetic deletions and the MMS perturbation on the phosphoproteomic and mRNA levels. At the outset, we confirmed the identities of the MCF10A wild type, MCF10A TP53 (-/-) knockout, and MCF10A PTEN (-/-) knockout cell lines by STR (Short Tandem Repeat) fingerprinting and CODIS (Combined DNA Index System) typing. The deletions of the TP53 and PTEN genes in the MCF10A knockout cell lines were also confirmed by RNA-seq. As illustrated in Fig. 4a, TP53 expression was significantly diminished in the TP53-knockout cell line compared to the MCF10A wild type and PTEN-knockout cell lines. Analogously, the same was true in the case of the PTEN-knockout cell line (Fig. 4b). The residual abundances observed for TP53 and PTEN are most likely due to only the partial genomic sequences having been removed. In contrast, when tested by Western Blotting, there is no evidence of p53 or PTEN protein expression in the TP53- or PTEN-knockout cell lines, respectively, when compared to the wild type cell lines (Fig. 4c,d). (Protease and phosphatase inhibitors were added to the cell line samples during the lysis step to conserve the proteomic and phosphoproteomic integrity of the samples.)

**Fig. 5:**
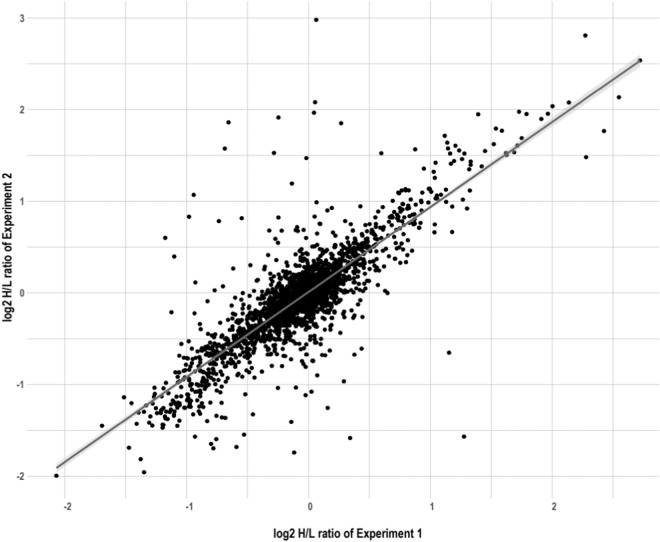
Phosphopeptides measured in SILAC biological replicate experiments are highly correlated. Differences in phosphorylation were measured in MCF10A-WT cells treated with MMS or mock-treated in biological replicate experiments (see Expts 1 and 2 in Table 1: MCF10A-WT-MMS (heavy)/MCF10A-WT-Mock (light)). Log(2) Heavy/Light ratios for phosphopeptides having localization scores > 0.8 are plotted using the MaxQuant-normalized ratios of the maximum MS1 peptide intensity for each peptide identification.

As control experiments for the phosphoproteomic data, we included label-swapping replicates such that heavy-SILAC-labeled cultured mammary cells were exposed to MMS in one experiment but mock-exposed in the replicate (Table 1). The concordance between the three forward and reverse experimental pairs (experiments 2 and 3, 4 and 5, and 8 and 9 in Table 1) was good with more than 75% of the data having <20% difference. In addition, two of the experiments were biological duplicates generated on two different days ((MCF10A-WT-MMS (heavy)/MCF10A-WT-Mock (light)), experiments 1 and 2 in Table 1), and the repeatability of the quantitative ratios for these two experiments was good, with a Pearson correlation coefficient of 0.828 (Fig. 5).

**Fig. 6:**
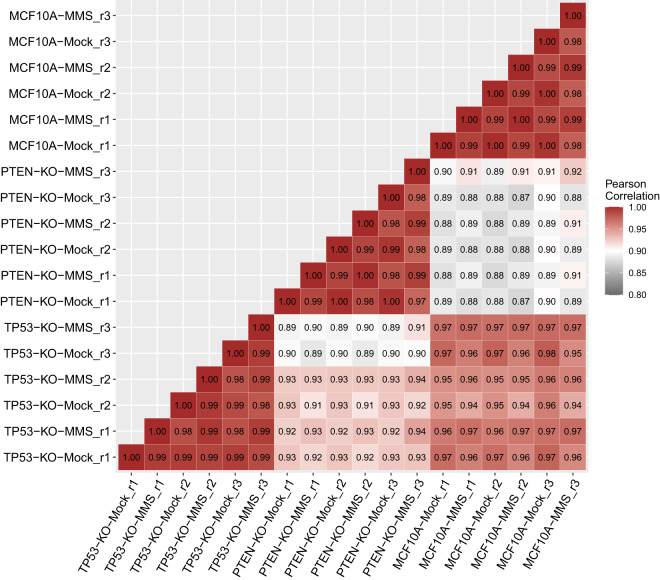
Repeatability of the RNA-seq data. The heatmap illustrates the correlation between pairs of RNA-seq samples. The legend indicates the strength of the correlation coefficient (red: high correlation; black: weak correlation).

For the RNA-seq analyses, three biological replicate samples were independently processed for each cell line and treatment condition, with the replicates spread over three different days (Table 2). To assess replicability of the sample preparation process, a pairwise heatmap plot was generated for the RNA-seq data (Fig. 6). There was good correlation (>0.98) among replicate RNA-seq profiles, and the average CV of the expression levels for three replicates was 13.3%.

**Fig. 7:**
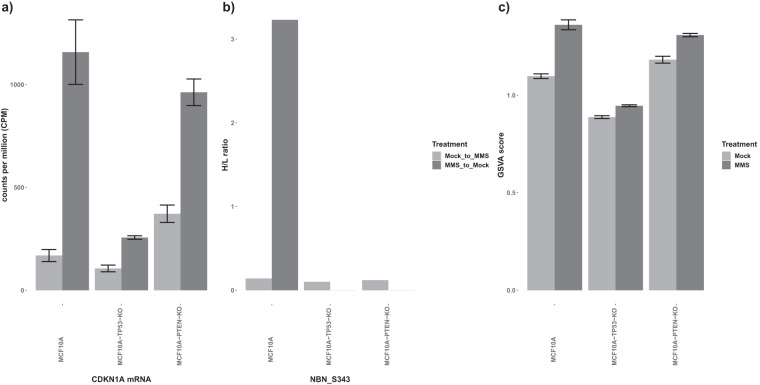
(**a**) CDKN1A RNA-seq gene expression across all three cell lines with MMS (dark grey) or Mock (light grey) treatment. Error bars indicate the standard deviation based on three biological replicates. (**b**) Nibrin (NBN) S343 relative phosphorylation levels across the three cell lines with or without MMS treatments (the data are based on experiments 2-3, 4-5, and 8-9 in Table 1). (**c**) Effect of TP53 deletion. Bar plot showing the increase in Gene Set Variation Analysis (GSVA)^24^ score with MMS treatment is significantly higher for MCF10A-WT compared to TP53- and PTEN-KO cell lines.

We performed further quality control analyses by assessing whether the RNA-seq and phosphoproteomic results were consistent with prior biological literature reports. For example, transcriptional upregulation of CDKN1A in response to MMS has been documented^22^. We evaluated whether this effect was corroborated by our data and found upregulation of CDKN1A gene expression across all cell lines with MMS perturbation (Fig. 7a). At the post-translational level, phosphorylation of the S343 site of nibrin (NBN) has been documented to be induced by DNA damage^23^. In our work, in response to MMS treatment, phosphorylation of this S343 site was also significantly increased in MMS-treated MCF10A-WT cells compared to the cells that received mock treatment (Fig. 7b).

To validate TP53 activity, we performed Gene Set Variation Analysis (GSVA)^24^ to evaluate a “wild type” TP53 signature based on the previously identified core TP53 transcriptional program by Andrysik *et al*.^10^. We focused on 31 key genes with direct binding that were identified in all three cell line experiments (HCT116, MCF7, SJSA)^10^, and obtained the single-sample Gene Set Enrichment Analysis (ssGSEA) scores for the wild type TP53 signatures in each cell line (Fig. 7c,Table ‘TP53 GSVA results’ in ‘Data and Results Summary Tables’ (data are at figshare)^17^). As expected, the WT TP53 signatures were higher in MCF10A than the MCF10A-TP53-KO samples. We also evaluated the significance of ssGSEA scores of the TP53 signature by comparing them with those from 1000 subsets of randomly selected genes with equal size. The p-value of TP53 signatures in MMS-treated MCF10A is 0.008 vs. 0.549 in Mock. On the other hand, p-values of TP53 signatures were not significant in either MMS-perturbated or Mock TP53-KO cell lines (p-value = 0.149 and 0.169, respectively) or in MMS-perturbated or Mock PTEN-KO cell lines (p-value = 0.185 and 0.183, respectively).

## Usage Notes

The identification and quantification results from the MaxQuant analysis can be downloaded from ProteomeXchange^16^ to be further interrogated. Also, the raw data files from the LC-MS/MS analysis of the phosphopeptide-enriched, SILAC labeled samples can be downloaded from the public repository^16^. These raw files can be analyzed by platforms other than MaxQuant, or they can be converted into an open data format (e.g., mzML, mzXML) to be compatible with even more proteomic data analysis platforms. Raw fastq files from GEO^18^ can be used as input for other downstream analyses such as to perform alternative transcript quantification analyses using Expectation Maximization (RSEM)^25^, to estimate differential gene expression with various statistical algorithms, and to explore enrichments in signaling pathways using differential gene lists.

Together, these data can serve the research community by potentially lending strength to genes and phosphopeptides that might be differentially observed with other chemical perturbations in similar experiments and datasets and by facilitating bioinformatic analyses of human cell or tissue ‘omic profiles.

## Data Availability

No custom code was used in this work. The R packages that were used to analyze the RNA-seq data are given in the methods section.
